# Assessment of Pain Onset and Maximum Bearable Pain Thresholds in Physical Contact Situations

**DOI:** 10.3390/s22082996

**Published:** 2022-04-13

**Authors:** Doyeon Han, Moonyoung Park, Junsuk Choi, Heonseop Shin, Donghwan Kim, Sungsoo Rhim

**Affiliations:** 1 Department of Mechanical Engineering, Kyung Hee University, Yongin-si 17104, Korea; hdy530@khu.ac.kr; 2 Department of Industry-Academic Cooperation Foundation, Kyung Hee University, Yongin-si 17104, Korea; mypark@khu.ac.kr; 3 Safetics, Seoul 07255, Korea; jschoi@safetics.io (J.C.); hsshin@safetics.io (H.S.); 4 Department of Physical Medicine & Rehabilitation, Kyung Hee University Hospital at Gangdong, Seoul 02447, Korea; kdhkjr@paran.com

**Keywords:** human–robot interaction, collision safety, collaborative application, biomechanical limitation, pain threshold

## Abstract

With the development of robot technology, robot utilization is expanding in industrial fields and everyday life. To employ robots in various fields wherein humans and robots share the same space, human safety must be guaranteed in the event of a human–robot collision. Therefore, criteria and limitations of safety need to be defined and well clarified. In this study, we induced mechanical pain in humans through quasi-static contact by an algometric device (at 29 parts of the human body). A manual apparatus was developed to induce and monitor a force and pressure. Forty healthy men participated voluntarily in the study. Physical quantities were classified based on pain onset and maximum bearable pain. The overall results derived from the trials pertained to the subjective concept of pain, which led to considerable inter-individual variation in the onset and threshold of pain. Based on the results, a quasi-static contact pain evaluation method was established, and biomechanical safety limitations on forces and pressures were formulated. The pain threshold attributed to quasi-static contact can serve as a safety standard for the robots employed.

## 1. Introduction

The robotics industry is one of the key sectors driving the Fourth Industrial Revolution and is essential for maintaining and strengthening manufacturing competitiveness [1]. Consequently, many countries are attempting to improve the manufacturing sector by fostering the robot industry and developing robot-related technologies [2]. According to World Robotics 2020 report published by the International Federation of Robotics, 2.7 million industrial robots were operating in factories in 2019. It indicates a 12% increase compared to the previous year. Concurrently, the installation of collaborative robots (CoBots), which collaborate with humans, is on the rise [3]. In addition, with the prolonged coronavirus disease 2019 (COVID-19) outbreak, companies are expected to use robots more actively to minimize the risk of infection stemming from face-to-face service [4].

Recent advances in robotics technologies show the possibilities for CoBots to link automated work cells and manual labor [5]. CoBots have the potential to enhance the capability of both robots and humans, while also increasing efficiency in the industrial, service, and medical fields [6]. Despite the potential merits and increasing need for their use, CoBots installed in industrial sites require substantial exertion to validate their risk and enhance their safety [7,8]. Therefore, regardless of the considerable development of sophisticated safety features, further enhancement in assessing robot safety, especially in physical contact situations between humans and robots, is still lacking and requires considerable attention [9].

ISO/TS 15066 provides the safety requirement for operating CoBots installed in industrial manufacturing sites [10]. The ISO standard defines two types of physical contact situations between robots and humans, transient and quasi-static [11]. This is important for robotic applications in which physical contact occurs or is likely to occur. Transient contact corresponds to situations where the operator immediately loses direct contact with the robot (for example, in instances wherein a certain part of the robot hits the body of the operator). Quasi-static contact is when the operator is clamped between the robot and a fixed object (for example, when the operator’s hand placed on a tabletop is pressed against a robot gripper) [12]. In either case, the safety of the user must be ensured. To formulate safety standards, technology is required for the evaluation of the safety of robots with respect to various injuries caused by collisions.

Some studies evaluated the degree of tissue injury through a collision experiment using tissue from pigs (with similar physical properties to humans) to confirm the safety standards for collision between robots and humans [13,14,15]. Most of these studies have analyzed the tendency of tissue destruction by impactor and contact force, contact pressure, and transferred energy. Although well representative, the use of pig tissue remains insufficient to authentically reflect the conscious reaction when accidents occur during robot–human interactions. Studies reporting the evaluation of new pressure pain thresholds as a measure of pain onset in humans have also been performed [16,17]. These pressure pain thresholds, along with other force pain thresholds, were reflected in ISO/TS 15066 and presented as biomechanical limitations [10]. These thresholds were applied to a virtual sensor as a safety limitation [18]. However, when applying the pain onset thresholds as biomechanical limitations in the power and force limit (PFL) mode, productivity may decrease due to the use of an overly conservative approach for safety. The PFL mode, one of the collaborative operation methods suggested by ISO/TS 15066, presupposes that the fundamentally safe operation of the robot should not exceed the biomechanical limit when the robot collides with a human. Therefore, since the concept of a physical or virtual safety fence is not required, it is relatively free from space constraints compared to other methods. This makes it easy to in-crease the operational flexibility of the robot. Nonetheless, the absence of a safety fence renders collision with workers always probable, so apparent biomechanical limitations for collision safety and methods to prevent exceeding those limitations must be prepared.

In this study, the thresholds for pain onset and maximum bearable pain were evaluated based on clinical trials. This procedure aims at establishing biomechanical limitations clearly and intuitively as robot collision safety criteria. We hypothesized that the obtained results could enable the formulation of improved biomechanical limitations over the current limitations, which are extremely restrictive in terms of efficiency. We carried out the application of quasi-static contact between human body parts and a contact probe to measure the force and pressure that cause pain onset and the maximum bearable pain. The clinical trial measured those contact forces and contact pressures for 29 human body parts. To compensate for the limited number of subjects in clinical trials, the representative values were recalculated by applying the inverse cumulative distribution function. Residual pain and skin injury after reaching the maximum tolerable pain were also analyzed through various criteria. The pressure and force derived from the thresholds for pain onset and maximum bearable pain can be regarded as safety limits and can serve as a reference standard for work safety.

## 2. Materials and Methods

### 2.1. Pain Threshold Assessment Apparatus

**Figure 1:**
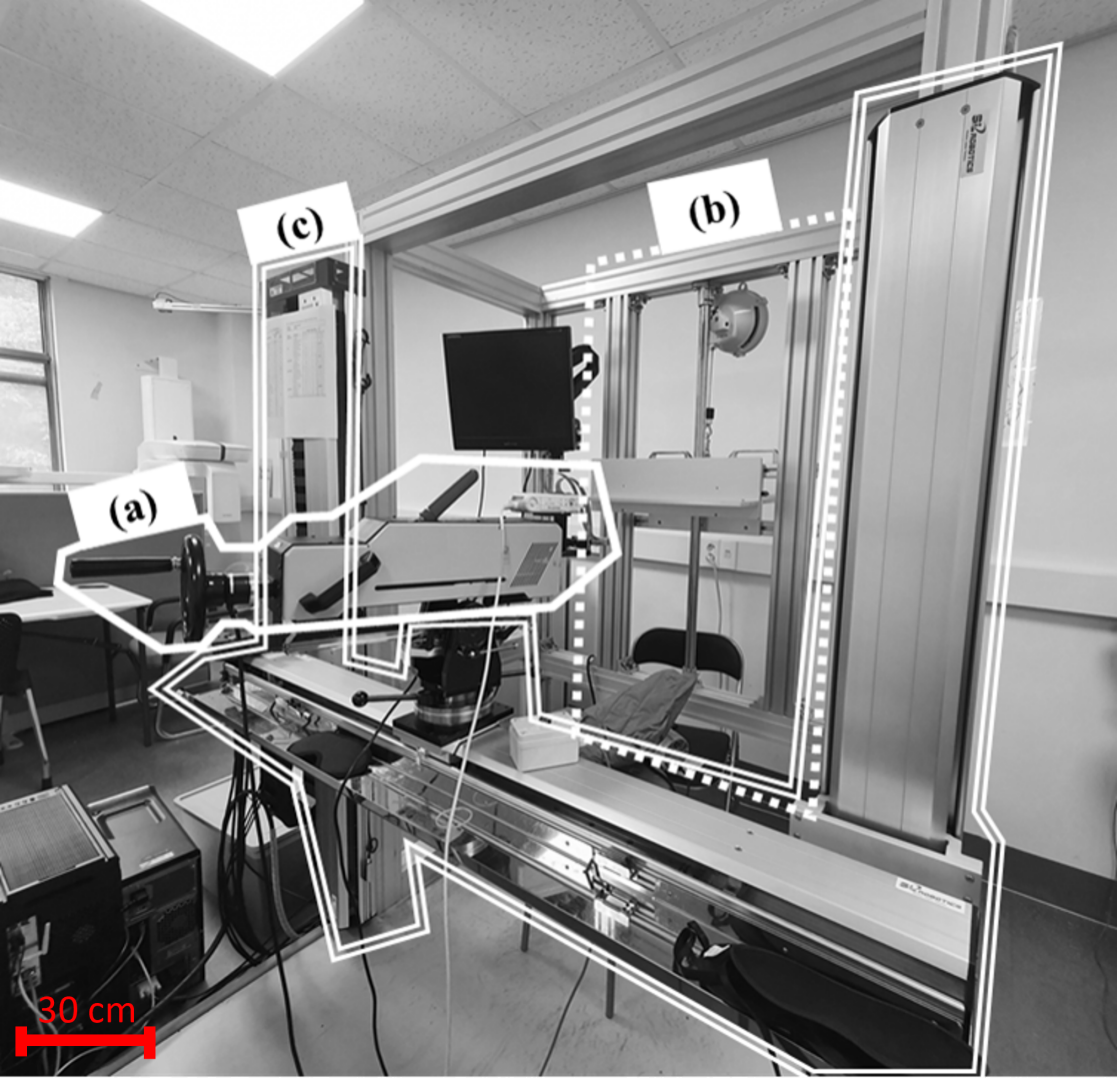
Test apparatus system consists of (**a**) algometer part, (**b**) stationary stanchion part, (**c**) algometer transfer and rotation system.

The pain thresholds were measured using a custom-made apparatus. The apparatus was referred to as a pain threshold measurement device, shown in Figure 1. Parts of the apparatus were developed to secure quantitative data on the pain felt by the subject through the quasi-static contact (clamping) at various body parts. The pain threshold measurement device consists of the algometer part, stationary stanchion part, and algometer transfer device. The algometer part is an instrument that operates based on a mechanism to measure the contact force and pressure data when a quasi-static contact occurs. It moves the contact probe along a directional axis perpendicular to the measurement site on the subject. The stationary stanchion is a device that holds the subject’s body part in a relaxed state so that it does not slide backward during the trial. The algometer transfer and rotation system is a device that translates about two axes and rotates about three axes, namely vertical and horizontal translation and rotation about the motion axes of the contact probe. Thus, the operator could position the contact probe in a vertical direction against the measurement site of the subject.

The algometer part comprises a sensor, contact probe, hand wheel, and safe retraction lever. This algometric device is capable of progressively increasing the load applied to subjects and measuring the distance of the contact probe. Low-velocity contact (contact duration over 0.5 s) was applied to simulate the quasi-static contact situation, as suggested in ISO/TS 15066. The contact probe is a part that contacts the body part. The hand wheel with a revolving handle is located on the opposite side of the contact probe. When the operator rotates the hand wheel manually in the clockwise direction, the contact probe part gradually moves forward. The movement direction of the contact probe was fixedly adjusted perpendicularly to the measurement point of the body part by the algometer translation and rotation system. In addition, the contact probe was carefully designed such that it did not slide even when a force was applied from the measuring unit. We consider this advantageous for safety as it is easier to respond to emergencies using a manual mechanism that employs a lever rather than an electric machine using a motor. A load cell and film pressure sensor were installed on the contact probe to measure contact force and contact pressure. The load cell had been calibrated beforehand, and the pressure was calculated through the force measured by the load cell.

**Figure 2:**
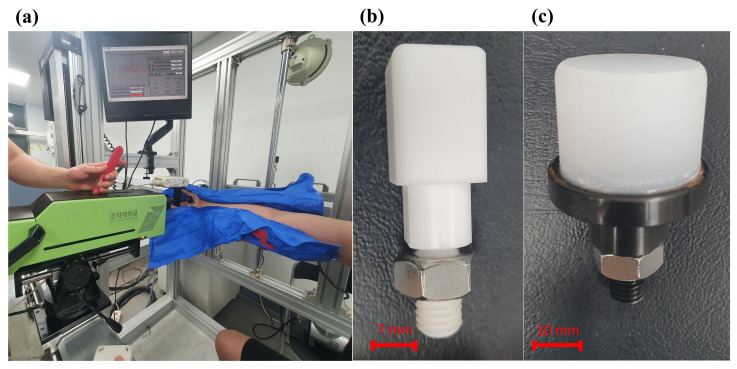
Elements of algometer part used in this study: (**a**) clinical trial with algometric system, (**b**) contact probe for measuring contact pressure, (**c**) contact probe for measuring contact force.

As shown in Figure 2, we used two types of contact probes; one has a shape that causes pain with increasing pressure and the other has a shape that causes pain with increasing force. The first contact probe has a small area such that it can be easily attached to the pressure film sensor, and the pain is not generated by force. The second contact probe has a large contact area such that no pain is caused by pressure. The part in contact with the measurement site is made of elastic rubber so that force is not applied only to a specific area during bending of the human body. The load cell (UMA-K50, Dacell, Cheangiu-Si, Chungcheong buk-Do, South Korea) measures forces along the direction of uniaxial load cell and has a permissible measured force of 500 N. The force sensor was equipped with a contact probe and attached to the end of the algometer. As a product of the I-Scan System (Pressure Mapping Sensor 4041, Tekscan, South Boston, MA, USA), a sensor capable of measuring the contact pressure was used by mounting a film-type pressure sensor. The sensor unit of the film-type pressure sensor was attached to a contact probe for measuring pressure.

Once the contact area of the probe established vertical contact with the target body part, the measuring device was configured to proceed vertically in the same direction. In addition, the position and direction of the pain meter were designed to be freely adjustable along three axes: abscissa, ordinate, and applicate. Furthermore, the measurement site was restrained in the same position and shape by fixing it with a locking device, and pain was induced through quasi-static contact. A supporting prop was added to fix the subject’s muscles in the measurement area without tension. According to the body size of the various subjects, the support was designed to have a structure that could be adjusted in the up, down, left, and right directions. As 29 selected body parts were measured, the support was easily transformed into different shapes. Therefore, it was necessary to use a vacuum cushion between the subject and stationary stanchion part. During measurements, the cushion helps fix the body part to avoid leaving any unnecessary space immediately behind it.

### 2.2. Human Subjects

Forty healthy male subjects aged 20–29 years participated in this study. Detailed information on the participants is listed in Table 1. All plans of this study were approved by the Clinical Trial Ethics Committee of Kyung Hee University (KHUH Institutional Review Board (IRB) File No. 2020-06-014). The protocol of the clinical trial was performed according to the guidelines and regulations of the IRB. Written informed consent was obtained from all the subjects who participated in the clinical trial. The following inclusion criteria were applied when selecting subjects:

1. Adults aged 19 to 59 years old who do not meet the exclusion criteria.
2. Adults who voluntarily agreed to the consent form for the clinical trial.

The exclusion criteria mentioned above are as follows:

1. Acute illness or serious psychological or mental problems
2. Chronic diseases that may interfere with the interpretation of therapeutic effects or outcomes (diabetes, hypertension, stroke, arrhythmia, ischemic heart disease, malignant tumor, allergic disease, nervous system disease, musculoskeletal disease, chronic obstructive pulmonary disease, asthma).
3. Insertion of artificial metal inserts in the body, such as artificial heart beaters, metal artificial joints, or prosthetics.
4. Students belonging to the College of Medicine, Kyung Hee University (undergraduate students, graduate students).
5. Students belonging to the Department of Mechanical Engineering, College of Engineering, Kyung Hee University (undergraduate students, graduate students).
6. Metal allergy, pain, or sensory abnormalities.
7. Other cases of acute infection or adverse drug reactions.
8. Presence of a wound or bruise at the pain measurement site (based on checking the case survey before the trial).
9. Illiterate.
10. In case pregnant or may become pregnant.

Basic physical data, including height, weight, body mass index (BMI), and circumference (upper arm, thigh, and shin), were collected from all participants, and verbal assessments of health status were conducted.

Because pain sensitivity can affect the overall experimental results, the subjects were tested when they were well rested and fed. We strongly recommended sleep for at least 6 h on the previous day. Of course, in practice, the various states of various workers could be estimated, but in this study, it was assumed that the state of not being hungry and taking adequate rest was the most frequent. Additionally, although subjective judgment about pain is unavoidable, to achieve similar measurement conditions for each subject, prior training was performed to enable the subjects to clearly understand the onset of pain and the maximum bearable pain. In the prior training, the experimenter sufficiently explained the concept of pain onset to all subjects. This explanation aimed at enabling them to understand the changing sensations when the feeling of a pressure or force on the skin is converted into pain. In addition, to confirm that the subjects actually understood the onset of pain, a quasi-static contact through a probe was performed on a site other than the target site of the actual trial. The experimenter identified the sensation felt by the subjects with questions and confirmed that they understood the exact onset of pain clearly. In the case of maximum bearable pain, the explanation was sufficiently detailed so that subjects could recognize that the maximum bearable pain level occurred when they felt that it was difficult to endure the pain anymore or felt like it could lead to injury if the trial continues.

In judging the pain onset and the maximum bearable pain, making them completely objective is difficult because there are individual differences in patience and sensitivity. To compensate for this subjectivity, quantitative criteria for pain, such as the Wong–Baker face pain rating scale and the numeric pain rating scale presented in Table 2, were applied to make the study as objective as possible [19,20]. The level of pain onset was quantified with a focus on matching the mild pain area of the numeric pain rating scale. The level of maximum bearable pain was also quantified based on matching the eighth grade of the Wong–Baker face pain rating scale by observing the subject’s face. It is also difficult to judge whether BMI reflects the thickness of the fat in each body region. Experimentally, it is almost impossible to measure the thickness of the fat and muscle in all body regions. Therefore, we measured the circumference of the arms, thighs, and calves in all subjects.

### 2.3. Pain Measurements

**Figure 3:**
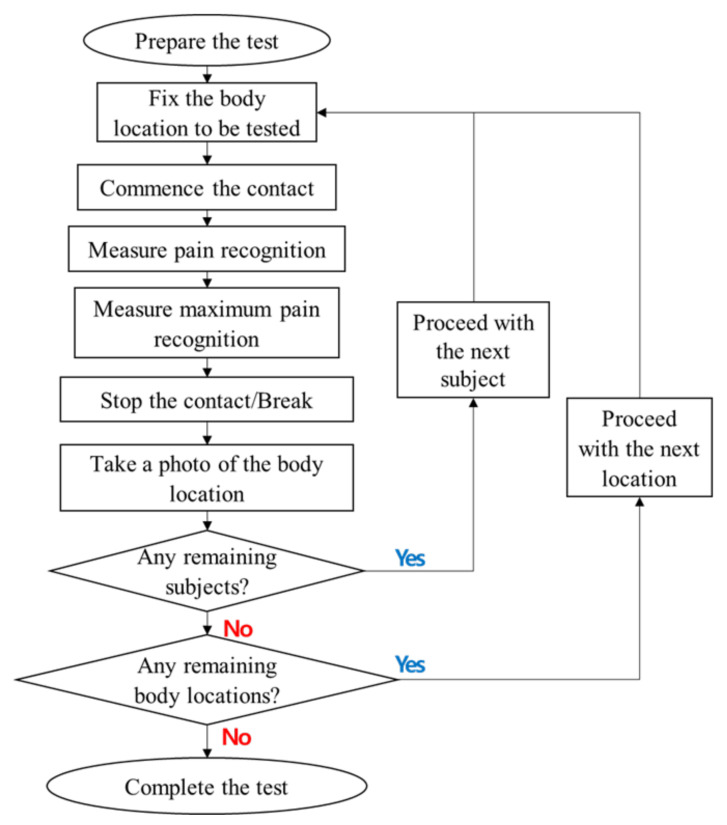
Flow diagram of clinical trial procedure.

**Figure A1:**
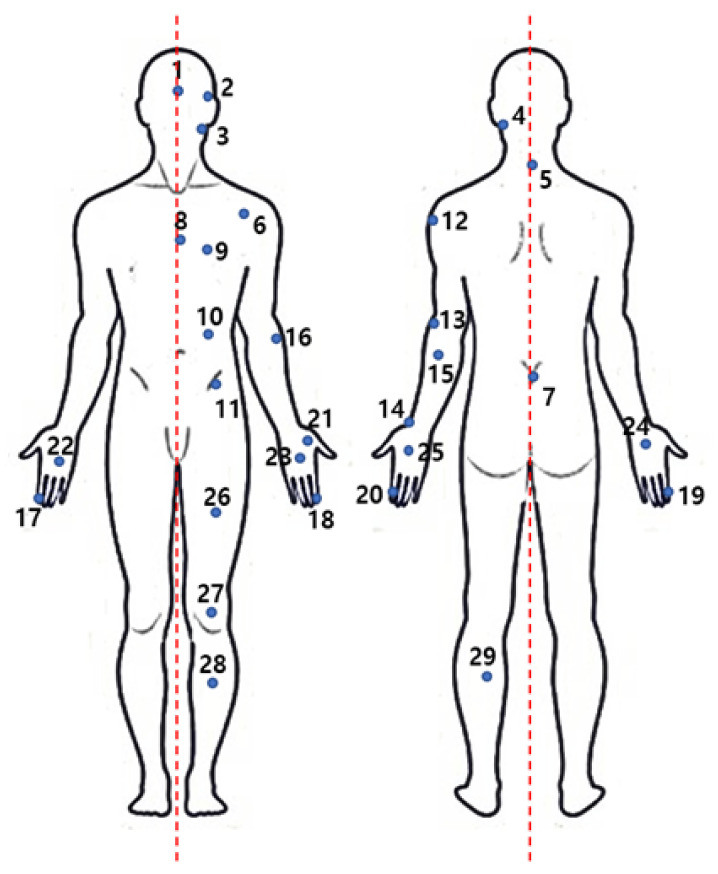
Measurement points and the numbering of each point.

All the body parts for measurement are shown in Figure A1 and Table 3, according to ISO/TS 15066. The red dashed line of Figure 3 marks the middle of the body to help confirm the exact position. In the second process, the first part was randomly selected at each visit of the subject. The sequence after the first part was randomly selected as the part furthest from the previous measurement part. (For example, 1. forehead (No.1) 2. thigh muscle (No.29) 3. deltoid muscle (No.12) …) Depending on the pain of the subjects, the order also changed within the group. The location of each measurement site was selected in consultation with a rehabilitation medical specialist to prevent additional reactions.

The subject was asked to press a button to measure and store the force, and the pressure was measured once at the start of the test. Then, the load causing the force and pressure was maintained, and it was stopped when the pain was unbearable. In this study, pain onset and maximum bearable pain were defined as follows.

- Pain Onset: The point at which a pressing feeling (pressure) is felt as pain (Wong–Baker face pain rating scale 2-4); this corresponds to the degree to which the robot can be used again as it is, despite the fact that the pain was felt after collision with the robot.
- Maximum Bearable Pain: The point in time at which pain can no longer be tolerated (Wong–Baker face pain rating scale 6–8); the degree of avoidance of stimulation by feeling strong pain after collision with the robot.

### 2.4. Data Collection Procedure

In Figure 3, the pain threshold was determined based on the following process. Pain recognition means the moment at which pain initiated, and maximum pain recognition corresponds to the moment of reaching maximum bearable pain.

1. First, the experimenter explained the purpose and method of the experiment to the subjects. The subjects understood the experiment and decided to participate.
2. The subjects received the necessary pre-training to conduct the exam based on the Wong–Baker face pain rating scale used in this study, the concept of pain onset and maximum tolerable pain, and the sequence of trial progression.
3. We then fixed the first part to the support according to the subject’s visits.
4. The first measurement was acquired at the moment at which contact was initiated and the subject felt a squeezing force (or pressure) as pain.
5. We continued to move the contact probe and acquired a second measurement when the subject felt the maximum bearable pain.
6. After the measurement was completed, we acquired a photograph of the measurement site.
7. This procedure was repeated for the next subject and for all the remaining body parts.

### 2.5. Residual Pain Questionnaire Assessment

In this study, residual pain was defined as the remaining pain on the skin after the subject’s measurement site was pressed, namely one of the sensations that remained for a while after the stimulus disappeared. We asked the subjects regarding residual pain at the measurement site 24–48 h after the collision event to determine whether any pain remained. If there was any pain, the subjects quantified the intensity of the residual pain. A specialist conducted a residual pain questionnaire assessment by pressing the measurement site. The subjects numerically expressed the extent to which they felt residual pain in the measurement area compared with the normal (non-measured) area.

For this numerical evaluation, residual pain at the measurement site was analyzed according to the pain evaluation methods shown in Table 2. The degree of residual pain remaining at the measurement site that interfered with daily life was defined as 4 (requiring a visit to the hospital) or higher. Additionally, we asked the specialist at the hospital treatment to provide treatment in case it was considered necessary. Furthermore, some subjects’ pains could not be clearly defined as an integer. Therefore, less intense pain was divided by units of 0.5 within the range of 0.5–3.5 points.

### 2.6. Skin Injury Evaluation

We marked the location of the measurement site based on the test sequence of the subject. Subsequently, the measurement sequence was conducted such that the skin surface of the measurement site was perpendicular to the compression direction. Further, the subjects were subjected to skin reaction measurements after the end of the trial. The skin surface at all the areas where the pain was measured was captured using a dermatology research camera and recorded as image data after at least 24 h. We analyzed the reaction on the skin surface based on the following two classifications:

- A skin reaction that caused the skin to turn red (with or without pain) in the subject’s experimental area after the trial.
- A capillary burst on the subject’s experimental skin surface, resulting in a speckle or petechia (with or without pain), defined as a vascular reaction.

Thus, the degree of skin injury was classified as either skin reaction severity or vascular reaction severity based on the grade. A detailed description of the grades is outlined in Table 4.

### 2.7. Energy Density

In some previous studies, energy density has been considered as a pain-causing factor [21]. In addition, in forensic science, energy density is considered as a factor responsible for the occurrence of contusion [22]. The equation used to obtain the energy density is as follows:

(1) $$e_{d}=\frac{\int_{s0}^{sf}F\;ds}{A_{contact}}$$

where $e_{d}$ denotes the energy density, $A_{contact}$ denotes the contact surface area, *F* represents the contact force, and $s_{0}$ and $s_{f}$ denote the distances of the probe at the start and end of the contact, respectively. Given that both the displacement of the probe and contact force were measured in this study, the energy density can be derived as shown in Equation (1). In the Results section, this energy density is compared with the force and pressure pain thresholds.

### 2.8. Statistical Analysis

A fitness test was performed to determine the distribution of pressure pain thresholds according to the body parts. A goodness-of-fit test was performed to determine the distribution of the pressure pain threshold according to the body site. Multivariate logistic regression analysis was performed to evaluate the relationship between pressure pain threshold, age, and BMI. Statistical analyses were conducted using SAS (version 9.4, SAS Institute Inc., Cary, NC, USA) and MATLAB (version, MathWorks, Inc., Natick, MA, USA). Statistical significance was set at *p* < 0.05.

## 3. Results

### 3.1. Descriptive Statistics

A descriptive statistical analysis of the pain onset and maximum bearable pain was conducted on all 29 body parts tested in the clinical trial. The results are listed in Table 5. The thresholds corresponding to pain onset and maximum bearable pain were divided by force and pressure. The measured pressure and force value distributions varied depending on the characteristics of body parts. The predicted value of the third quartile in Table 6 was calculated by the inverse cumulative distribution function. Because the procedure of calculating the inverse cumulative distribution function entails distribution estimation, the results could compensate for the limited number of samples of 40. In Table 5 and Table 6, “pain tolerance” indicates the threshold for maximum bearable pain.

**Figure 4:**
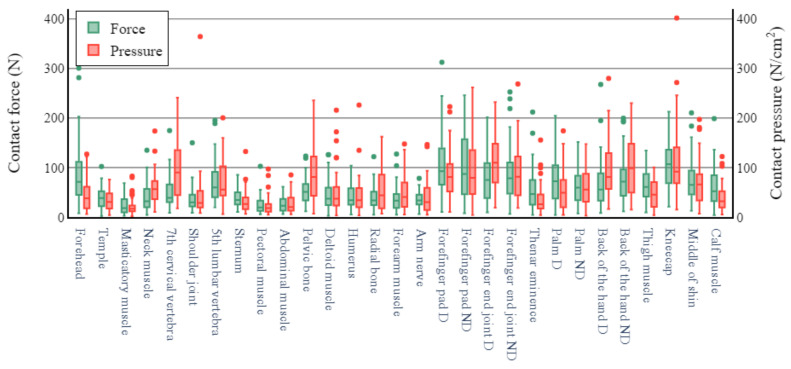
Pain thresholds for pain onset.

**Figure 5:**
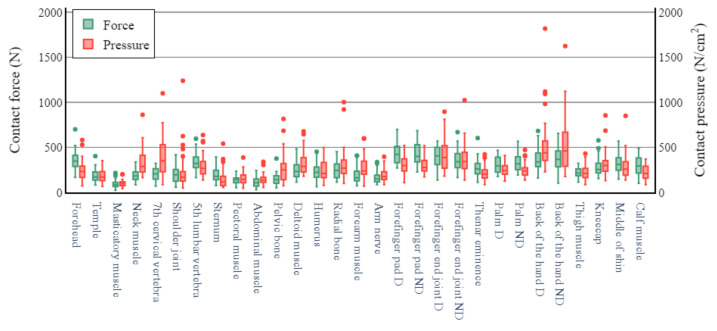
Pain threshold for maximum bearable pain.

The pressure and force thresholds that initiated pain are depicted in Figure 4 for all measurement sites. The pressure and force thresholds corresponding to the maximum bearable pain for all measurement sites are shown in Figure 5. The pressure pain thresholds varied extensively among the measurement sites. In Figure 4,the joint, muscle, and nerve sites yielded relatively low thresholds in the range of 53–148 N/cm^2^. Maximum bearable pain thresholds in the range of 250–410 N/cm^2^ were observed at the forehead, neck muscle, ball of the thumb, and shin. In the same category, relatively high thresholds (i.e., 570–670 N/cm^2^) were observed at the sites on the back of the hand. The lowest pressure pain threshold for maximum bearable pain was 120 N/cm^2^ at the masticator muscle, and the highest was 670 N/cm^2^ at the posterior part of the non-dominant (ND) hand. The pressure pain thresholds yielded a variety of pattern distributions at all the body sites. The mean threshold values and percentile ranges for all the measurement sites are listed in Table A1, Table A2, Table A3 and Table A4 in the Appendix A.

At the pressure pain threshold for maximum bearable pain, the pressure values exhibited smaller variations in the case of the contact probe comprising the hard material with hexahedral geometry (contact probe for measuring contact pressure) compared to those in the case of the soft probe with cylindrical geometry (contact probe for measuring contact force). In addition, the median force values were distributed in narrow ranges in the case of the arm or spine. However, there was a large deviation in the cylindrical shape of the soft material at the hands and legs.

**Figure 6:**
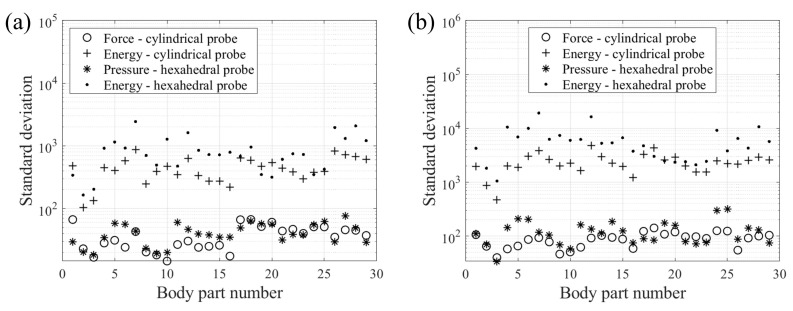
Standard deviation from the pain onset (**a**), and maximum bearable pain (**b**) at different body parts.

In addition, standard deviations for each body part were derived and compared to determine the most suitable parameters for the safety criteria. The graph on the left in Figure 6 represents the results obtained after the calculation of the standard deviation of the force, pressure, and energy at the onset of pain. The graph on the right depicts the results of maximum bearable pain. In some ordinary units, the standard deviation of the contact force was found to be the smallest in most cases. In this study, the energy density was calculated using a simple approach. In collision situations, because the transferred energy between robots and humans depends on the type of collision and there are various models and theories applicable in this regard, it is difficult to predict the exact transferred energy. Although this error is included, it is predicted that force and pressure could be more distinct indicators when observing the distribution based on the standard deviation of transmitted energy, contact force, and contact pressure.

### 3.2. Pain Thresholds

Table 5 lists the representative values of the measured force and pressure, divided by the pain on-set and maximum bearable pain. According to related literature and ISO/TS 15066, the third quartile is a representative value suitable for judging biomechanical limitations. The results for pain onset were lower than the biomechanical limit values of the PFL mode presented in ISO/TS 15066. The results for maximum bearable pain were found to exceed the standard ISO/TS 15066 values. In the case of pressure, considering that ISO/TS 15066 specifies the standard onset of pain, the results of this study were relatively different from those presented in the standard document. Additionally, after the degrees of injury were evaluated in subjects who reached the pain limit, it was confirmed that minor bruising occurred only in very few subjects.

### 3.3. Pressure Pain Thresholds

Statistics were analyzed to determine whether age, BMI, and circumferences of the arm, thigh, and calf affected the onset of pain and the maximum allowable pain in each region. Age contributes to the susceptibility of skin to trauma [23]. Based on BMI, the participants were divided into two groups according to a cutoff of 23 kg/m^2^, which is known to increase with the incidence of various geriatric diseases like type 2 diabetes, cardiovascular (heart and blood circulation) disease [24]. Correlation of these factors in results of the analysis by dividing the onset of pain and maximum tolerable pain showed a similar tendency to that of previous studies [17].

### 3.4. Skin Injury Evaluation Analysis

**Figure 7:**
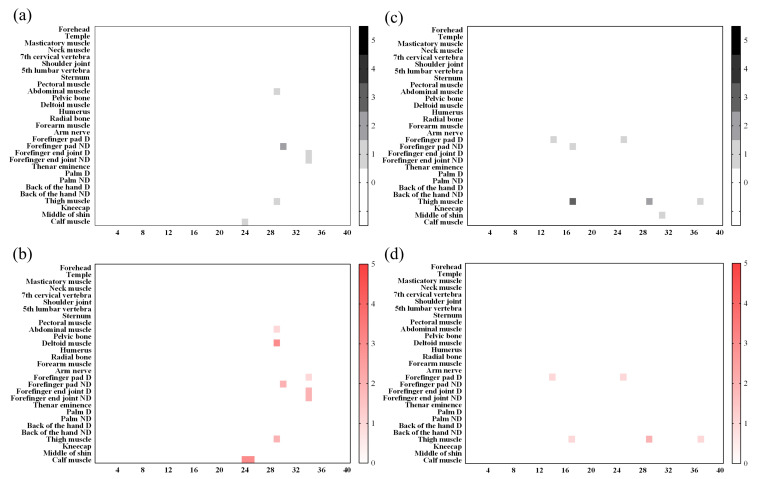
Analyzed heat map results for all subjects. From left to right, the results of the skin reaction (**a**), skin reaction (**b**), vascular reaction of the cube-shaped collider (**c**), and vascular reaction (**d**) of the cylindrical collider.

**Figure 8:**
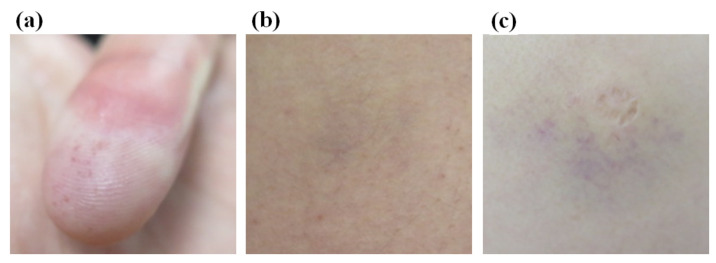
Representative photographs acquired to analyze the degree of skin and vascular reactions. (**a**) Skin reaction degree 2 (mild erythema)/vascular reaction degree 1 (petechia), (**b**) Skin reaction degree 3/vascular reaction degree 3 (purpura), (**c**) Skin reaction degree 0/vascular reaction degree 3 (purpula).

Figure 7 is a heat map graph showing the results according to the degrees of the skin and vascular reactions at different body parts after impact. All 29 body sites were measured up to the maximum bearable pain. A small number of subjects showed symptoms such as redness or bruising of the skin surface, and there were rare cases requiring hospital treatment. Those cases were formal classifications based on Table 2 and Table 4. However, by the diagnosis from the specialist, none of the subjects actually needed hospital treatment. There were no grade 3 (severe erythema, papular dermatitis, or violaceous purpura) or higher outcomes in skin and vascular reactions, which were both clinically evaluated. The rate of occurrence of “temporary faint erythema” of the cube-shaped (hexahedral) impactor was higher among the skin reactions. The vascular response rate was also high in the cube-shaped impactor. This result is observed more frequently when an impactor is made of a hard material rather than a soft material. In Figure 8, example photographs showing skin and vascular reactions analysis are depicted.

### 3.5. Residual Pain Questionnaire Analysis

**Figure 9:**
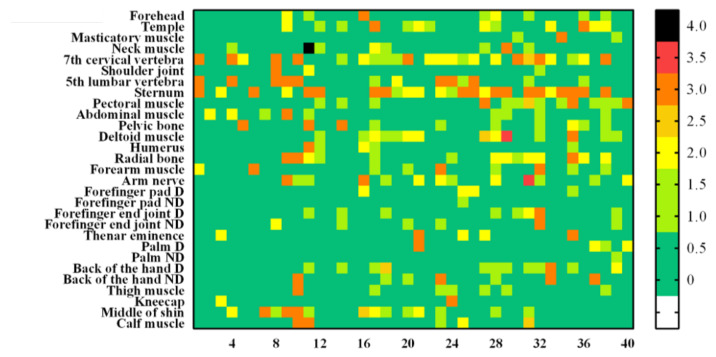
Residualpain after the testing of all 29 pain measurement sites (rigid hexahedral contact probe).

**Figure 10:**
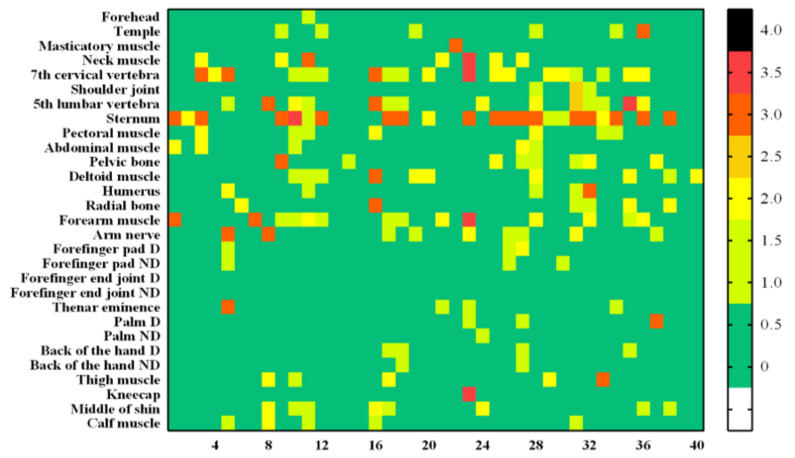
Residual pain after the testing of all 29 pain measurement sites (soft cylindrical contact probe).

As shown in Figure 9 and Figure 10, the level of residual pain was higher in the cylindrical impactor experiment. Additionally, subjects felt the pain threshold later in the case of the soft cylindrical contact probe compared to the rigid hexahedral contact probe. Although the same person measured the pain threshold at the same measurement site based on the same test protocol, the change in the shape and material of the impactor appeared to affect the results. In addition, residual pain was observed in more areas in the case of the hexahedral impactor than in the case of the cylindrical collider.

For both contact probes, most subjects experienced residual pain in the spinous process C7 and the sternum. In both areas, wherein there was a thin skin layer and the proportion of subcutaneous fat absorbing shock was low, mechanical force or pressure directly affected the skin layer and bone and caused residual pain. However, the pain levels were lower than the level that interfered with daily life, and none of the subjects felt any pain unless they deliberately pressed the measurement site.

## 4. Discussion

The main purpose of this study was to redefine safety criteria that determine the close interactions of robots with humans by quantifying the forces and pressure that cause the onset of pain and the maximum bearable pain in collisions with robots. Clinical trials were completed on 29 body parts in 40 men to determine the thresholds for the onset of pain and maximum bearable pain. Force pain thresholds relevant to the onset and maximum bearable pain caused by human–robot collisions were determined. Force pain thresholds relevant to pain onset were shown to be in the range of 23.7 ± 16.7 to 106.9 ± 66.4 N, and force pain thresholds relevant to maximum bearable pain were shown to be in the range of 90.4 ± 40 to 429.7 ± 141.8 N. Pressure pain thresholds relevant to pain onset that were caused by potential human–robot collisions were also determined. Pressure pain thresholds relevant to pain onset were shown to be in the range of 22.2 ± 18.3 to 113.1 ± 76.4 N/cm^2^, and pressure pain thresholds relevant to maximum bearable pain were shown to be in the range of 100.3 ± 34.2 to 529 ± 316.8 N/cm^2^. The range of biomechanical limits varied with individuals and measurements, age, and BMI. Pain is a highly subjective concept that can vary according to individual sensitivity and experience. Therefore, the subjects were controlled to avoid being affected by their physical condition (fatigue) and hunger. In addition, the measurement order was randomized to rule out the assertion that it depends on experimental factors such as the measurement order and time.

Collectively, when the pain thresholds for pain onset and maximum bearable pain were compared, the pain threshold for maximum bearable pain was clearly higher for all measurement sites. Furthermore, although the allowable contact force or contact pressure was increased, skin injury or residual pain rarely occurred. Considering these characteristics, if a safety criterion based on maximum bearable pain rather than pain onset is applied for safe trajectory generation or to the virtual sensor technology of CoBots, it is expected that productivity can be increased with relatively diminutive risk. Such a safety criterion would be applicable not only in industrial sites but also in various other environments.

## Figures and Tables

**Table 1:** Subject characteristics.

Characteristics	Description
Number of participants	40
Sex	Male
Age	20 to 29 years
Weight	72.0 ± 12.15 kg
Height	173.6 ± 5.80 cm
Body mass index (BMI)	23.82 ± 3.31
Mood	Good to normal
Arm circumference	29.14 ± 4.95 cm
Thigh circumference	48.75 ± 5.25 cm
Calf circumference	37.45 ± 3.41 cm

**Table 2:** Two of the generally used clinical pain assessment tools. We have used these two terms to describe the level of pain to the subject.

Pain Assessment Tools	
Scale	Image
Wong–Baker face pain rating scale	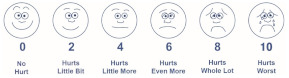
Numeric pain rating scale	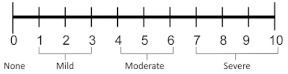

**Table 3:** Measurement points in each body part.

No	Body Part	Measurement Point
1	Skull and forehead	Middle of forehead
2		Temple
3	Face	Masticatory muscle
4	Neck	Neck muscle
5		Seventh neck vertebra
6	Back and shoulder	Shoulder joint
7		Fifth lumbar vertebra
8	Chest	Sternum
9		Pectoral muscle
10	Abdomen	Abdominal muscle
11	Pelvis	Pelvic bone
12	Upper arms and elbow joints	Deltoid muscle
13		Humeral bone
14	Lower arms and wrist joints	Radial bone
15		Forearm muscle
16		Arm nerve
17	Hands and fingers	Forefinger pad D ^1^
18		Forefinger pad ND ^1^
19		Forefinger end joint D ^1^
20		Forefinger end joint ND ^1^
21		Thenar eminence
22		Palm D ^1^
23		Palm ND ^1^
24		Back of the hand D ^1^
25		Back of the hand ND ^1^
26	Thighs and knees	Thigh muscle
27		Kneecap
28	Lower legs	Middle of shin
29		Calf muscle

^1^ D: Dominant side, ND: Non-dominant side.

**Table 4:** Criteria for grading the severity of skin reactions and vascular reactions at all the measurement body sites.

Skin Reaction Severity Score (SRSS)		Vascular Reaction Severity Score (VRSS)	
Grade	Criteria/Characteristics	Grade	Criteria/Characteristics
0	No reaction	0	No reaction
1	Transient faint erythema	1	Petechia-asymptomatic ^1^
2	Transient moderate erythema	2	Prickling petechia-symptomatic ^1^
3	Severe erythema or papular dermatitis	3	Violaceous purpura
4	Abrasion	4	Nontender ecchymoses
5	Scar	5	Tender ecchymoses

^1^ Analysis of “Symptomatic/Asymptomatic” is based on residual pain outcome.

**Table 5:** Representative values of pain onset and maximum limit owing to quasi-static contact at each part (third quartile by descriptive statistics).

Body Point	Pain Onset Force [N]	Pain Tolerance Force [N]	ISO Force Limits [N]	Pain Onset Pressure [N/cm^2^]	Pain Tolerance Pressure [N/cm^2^]	ISO Pressure Limits [N/cm^2^]
1	111.4	412.7	130	61.0	291.7	130
2	52.0	226.2	130	47.8	231.6	110
3	36.3	110.4	65	23.3	117.9	110
4	56.7	226.3	150	72.8	411.5	140
5	66.0	262.9	150	135.2	529.8	210
6	45.2	254.5	210	53.1	230.5	160
7	91.3	393.5	210	102.4	342.4	210
8	49.9	246.2	140	39.4	179.0	120
9	33.3	159.5	140	26.1	193.0	170
10	36.6	148.8	110	39.4	167.3	140
11	66.6	178.1	180	122.2	322.3	210
12	59.4	306.9	150	60.6	387.2	190
13	58.0	280.6	150	58.6	332.4	220
14	51.2	314.8	160	85.9	358.9	190
15	45.3	236.1	160	69.9	345.9	180
16	45.3	190.6	160	58.9	228.2	180
17	138.7	509.9	140	107.1	370.9	300
18	156.9	529.0	140	135.1	355.3	270
19	108.9	499.3	140	147.9	518.0	280
20	110.1	430.6	140	122.3	445.9	220
21	74.9	320.4	140	45.5	250.2	200
22	104.4	391.2	140	75.0	295.9	260
23	83.5	395.0	140	87.5	273.4	260
24	86.9	437.3	140	129.3	571.6	200
25	96.0	460.0	140	148.0	667.6	190
26	87.1	261.0	220	70.8	266.8	250
27	135.8	323.4	220	140.7	352.2	220
28	93.9	384.1	130	86.5	340.9	220
29	84.4	382.3	130	52.2	289.1	210

**Table 6:** Representative values of pain onset and threshold owing to quasi-static contact for each part (third quartile based on the inverse cumulative distribution function).

Body Point	Pain Onset Force [N]	Pain Tolerance Force [N]	ISO Force Limits [N]	Pain Onset Pressure [N/cm^2^]	Pain Tolerance Pressure [N/cm^2^]	ISO Pressure Limits [N/cm^2^]
1	112.4	413.8	130	54.3	307.8	130
2	54.6	217.3	130	44.9	221.4	110
3	30.9	111.4	65	28.5	118.7	110
4	53.1	222.4	150	75.2	402.3	140
5	62.5	243.4	150	130.0	502.8	210
6	44.4	247.4	210	52.8	281.6	160
7	90.1	387.3	210	91.0	345.2	210
8	49.7	234.7	140	39.0	184.9	120
9	31.0	164.7	140	27.8	190.8	170
10	32.3	141.3	110	35.3	174.8	140
11	66.9	173.7	180	120.1	329.1	210
12	60.3	300.8	150	65.5	390.1	190
13	54.3	296.4	150	57.0	305.5	220
14	52.0	301.0	160	66.6	385.8	190
15	46.4	226.4	160	63.5	338.9	180
16	46.6	190.4	160	51.9	229.2	180
17	141.7	496.3	140	113.2	359.7	300
18	134.4	511.4	140	131.1	343.4	270
19	106.0	467.0	140	144.4	498.3	280
20	120.6	429.8	140	117.3	443.7	220
21	73.9	324.9	140	46.1	252.2	200
22	101.8	366.9	140	70.4	297.4	260
23	89.2	376.3	140	84.6	291.5	260
24	85.0	435.9	140	122.3	634.6	200
25	102.6	437.6	140	135.5	664.1	190
26	85.0	254.3	220	63.9	268.6	250
27	131.2	323.0	220	145.2	375.4	220
28	98.5	380.9	130	94.8	337.0	220
29	79.0	360.6	130	51.1	269.5	210

## Data Availability

All data are available in the main text or the Appendix A.

## Appendix A

**Table A1:** Descriptive statistics on pressure pain thresholds at which pain onset occurs (N = 40).

Body Point	Pressure Pain Threshold for Pain Onset [N/cm^2^]										
	Mean	STD	MIN.	P5	P10	Q1	Q2	Q3	P90	P95	Max.
1	41.65	29.45	6.21	7.27	9.37	17.81	37.81	61.04	78.53	104.10	127.06
2	33.97	20.28	3.74	6.21	10.44	17.41	30.70	47.81	62.41	70.28	75.88
3	22.21	18.31	1.89	3.73	6.64	11.76	16.84	23.27	47.55	66.57	82.58
4	57.92	34.11	10.21	13.82	14.89	36.36	56.57	72.83	102.6	119.7	173.67
5	100.14	58.17	17.56	25.02	33.64	44.43	90.06	135.17	194.83	208.43	241.01
6	44.64	56.49	8.35	10.71	13.58	19.55	28.28	53.12	77.25	85.69	364.39
7	68.24	43.59	5.84	10.49	15.34	43.39	55.41	102.42	120.35	154.6	200.32
8	30.95	23.27	6.56	8.44	9.98	15.93	25.56	39.44	52.19	73.46	132.36
9	22.72	19.36	4.74	7.67	9.41	10.93	17.93	26.05	43.69	72.2	96.96
10	27.86	19.83	5.56	8.17	8.66	13.8	20.19	39.35	58.14	69.31	85.09
11	91.1	60.12	7.08	21.22	25.07	43.01	80.76	122.23	198.68	214.83	235.48
12	51.47	46.28	3.35	9.08	12.92	23.13	38.12	60.58	119.83	162.98	215.72
13	44.56	39.11	3.44	10.78	11.21	19.87	34.26	58.6	72.95	109.32	226.13
14	51.31	38.29	6.86	9.53	11.92	17.76	44.03	85.85	96.03	127.2	162.44
15	48.75	34.8	5.14	10.16	12.12	20.53	40.32	69.89	96.85	124.15	147.54
16	40.55	34.86	4.83	5.49	7.75	14.33	30.24	58.94	86.83	117.78	146.4
17	87.92	48.67	10.15	27.81	35.35	51.75	80.9	107.1	146.55	193.49	222.86
18	95.56	63.83	3.23	12.07	24.32	46.59	79.12	135.13	187	220.09	261.68
19	109.71	57.13	18.94	21.87	29.65	69.86	109.91	147.93	185.94	212.49	231.93
20	90.84	55.79	18.47	20.69	28.2	43.95	81.39	122.25	161.08	193.87	268.65
21	36.38	31.53	3.92	6.66	9.62	15.11	25.23	45.47	81.73	102.32	155.46
22	53.44	38.76	4.43	9.68	12.05	19.99	48.98	75	103.55	131.23	173.99
23	60.31	38.29	2.85	4.86	9.28	31.08	55.44	87.47	111.21	125.02	147.23
24	95.04	55.31	16.32	22.76	34.21	56.74	81	129.3	165.31	194.29	279.89
25	103.51	61.95	15.1	22.59	28.32	49.4	98.76	148	196.5	221.47	229.62
26	47.68	29.35	3.97	8.01	12.11	20.49	44.98	70.75	93.06	97.05	100.47
27	113.06	76.43	14.91	25.85	36.61	68.22	91.83	140.66	207.75	258.72	401.8
28	71.21	49.21	6.93	9.77	13.87	33.72	65.75	86.5	145.64	178.86	197.16
29	39.39	28.89	5.13	6.55	10.29	19.15	32.03	52.15	77.36	105.34	122.17

**Table A2:** Descriptive statistics on pressure pain thresholds that cause maximum bearable pain (N = 40).

Body Point	Pressure Pain Threshold for Maximum Bearable Pain [N/cm^2^]										
	Mean	STD	MIN.	P5	P10	Q1	Q2	Q3	P90	P95	Max.
1	250.81	111.59	70.95	125.24	131.87	166.15	232.01	291.66	392.49	465.6	581.68
2	182.06	70.6	67.15	90.93	95.28	129.42	169.41	231.61	276.18	316.26	352.09
3	100.27	34.17	41.18	61.52	64.6	73.51	96.2	117.86	139.44	172.5	200.61
4	333.69	143.52	163.71	166.21	196.89	226.13	288.05	411.51	496.29	575.12	861.31
5	391.12	210.59	85.41	88.86	140.05	229.23	350.61	529.81	612.71	719.62	1099.2
6	230.24	206.9	48.37	62.23	97.88	125.22	168.42	230.45	441.15	574.96	1238.8
7	287.54	118.06	136.69	145.27	165.24	205.44	267.58	342.35	451.78	563.04	636.54
8	148.52	104.36	36.24	55.55	60.18	75.97	115.79	179.02	299.67	354.97	541.13
9	155.5	68.54	45.45	66.56	85.42	108.13	144.46	192.96	241.6	279.44	386.85
10	144.43	58.22	54	60.53	79.86	111.63	137.56	167.27	209.75	266.22	339.49
11	262.99	161.16	73.99	97.52	103.98	141.2	249.26	322.31	459.56	612.33	815.71
12	320.11	136.25	81.52	184.43	195.4	225.56	282.45	387.2	545.55	610.38	677.16
13	246.83	114.12	77.55	96.22	133.54	158.48	199.69	332.35	425.89	478.69	497.09
14	311.27	184.17	72.33	106.54	146.02	208.82	269.55	358.88	478.53	710.3	1001.58
15	270.67	126.15	69.73	88.79	119.63	201.91	237.08	345.92	437.97	539.39	599.84
16	191.28	74.36	86.25	93.85	119.72	140.39	173.45	228.23	310.2	344.47	395.79
17	303.04	90	107.86	158.02	197.89	236.94	298.57	370.9	430.66	448.84	519.88
18	292.63	84.82	110.21	185.2	196.15	236.31	277.47	355.32	412.2	431.86	519.65
19	409.06	175.26	180.88	198.9	210.27	269.25	387.4	518.01	592.4	785.7	895.53
20	368.11	157.37	137.09	177.18	201.24	264.44	341.47	445.9	522.49	608.31	1023.57
21	208.56	79.87	86.53	98.74	115.57	155.41	203.38	250.19	324.52	373.8	422.23
22	254.64	72.12	125.46	140.78	163.94	202.47	242.5	295.9	370.66	396.48	410.28
23	249.34	76.67	135.72	137.32	163.3	193.46	238.95	273.38	361.68	405.76	470.79
24	523.88	298.49	229.71	247.14	262.88	356.01	438.19	571.61	874.21	1105.65	1817.93
25	529.02	316.81	126	188	218.04	290.25	460	667.59	1018.14	1090.5	1623.49
26	220.12	88.3	86.55	93.15	108.02	163.1	210.39	266.77	361.96	388.03	430.48
27	310.89	140.86	129.88	149.5	170.86	230.56	290.02	352.17	459.31	596.11	854.23
28	279.19	130.73	122.12	140.76	151.96	189.99	257.37	340.91	394	472.65	849.65
29	224.11	75.22	86.92	110.79	142.26	156.86	209.26	289.1	330.46	339.47	369.63

**Table A3:** Descriptive statistics on the force pain threshold at which pain onset occurs (N = 40).

Body Point	Force Pain Threshold for Pain Onset [N]										
	Mean	STD	MIN.	P5	P10	Q1	Q2	Q3	P90	P95	Max.
1	86.57	66.74	7.72	15.56	20.06	44.7	71.13	111.44	181.23	242.12	300.59
2	38.7	22.84	2	3.33	6.19	22.05	38.23	52.04	70.44	75.64	102.07
3	23.65	16.66	2.72	4.17	6.13	10.21	18.2	36.25	45.54	51.84	68.45
4	40.12	28.15	3	6.08	11	19.81	31.69	56.72	78.66	92.32	134.9
5	49.35	31.18	8.48	13.89	20.01	30.31	38.68	65.98	85.35	101.23	174.52
6	35.86	24.11	8.51	12.86	16.95	21.55	29.67	45.15	58.74	69.59	149.92
7	70.98	42.92	19.85	20.6	23.91	39.89	59.8	91.34	124.46	167.91	195.19
8	39.82	20.26	10.3	16.74	18.2	25.57	34.6	49.89	73.44	81.92	85.22
9	24.74	18.14	5.4	8.42	9.21	12.46	19.51	33.28	44.04	54.75	102.46
10	25.21	14.51	5.87	7.22	8.19	13.55	22.75	36.59	44.85	53.73	61.19
11	52.7	26.67	11.8	15.11	21.78	32.45	50.98	66.63	84.51	109.17	123.06
12	44.98	30.29	2.54	7.51	10.68	26.07	37.09	59.38	85.04	117.98	125.92
13	41.58	24.02	4.97	8.85	14.11	25.05	34.14	57.97	78.91	83.72	103.71
14	39.56	24.98	4.61	6.26	13.72	24.14	33.58	51.19	78.57	84.01	121.92
15	36.04	25.93	4.72	7.76	9.19	18.69	32.24	45.31	65.18	92.02	127.21
16	36.04	17.42	6.94	8.05	12	25.52	33.4	45.28	59.69	63.93	78.07
17	106.86	66.39	10.48	15.77	33.13	65.34	92.36	138.69	198.81	227.68	312.46
18	99.79	66.84	7.8	11.46	20.72	45.9	86.96	156.93	196.61	218.37	245.55
19	80.26	51.65	9.55	16	17.86	33.85	74.71	108.9	154.96	189.4	201.42
20	90.57	60.57	6.44	14.16	22.8	47.33	78.32	110.06	175.13	228.78	252.56
21	56.83	43.82	4.11	12.12	14.13	25.09	46.55	74.94	110.51	147.77	211.93
22	74.93	46.76	4.4	11.21	15.56	37.27	72.19	104.41	136.94	161.2	204.53
23	66.21	40.19	7.12	7.62	19.01	33.9	59.37	83.52	130.37	145.54	151.53
24	66.25	51.01	8.12	12.55	19.6	33.33	55.31	86.87	129.46	167.84	267.69
25	79.11	50.93	11.62	19.29	21.92	43.09	70.18	96.01	158.63	193.26	199.56
26	64.97	35.03	9.41	12.5	21.89	41.22	60.51	87.06	122.68	132.72	134.43
27	102.56	45.7	21.24	26.86	43.83	68.41	106.57	135.81	163.08	183.36	212.61
28	76.83	44.95	12.84	16.77	34.15	45.54	65	93.86	146.11	172.18	210.36
29	61.14	37.23	7.76	15.61	22.96	33.12	51.89	84.38	100.8	121.5	198.7

**Table A4:** Descriptive statistics on force pain thresholds that cause maximum bearable pain (N = 40).

Body Point	Force Pain Threshold for Maximum Bearable Pain [N]										
	Mean	STD	MIN.	P5	P10	Q1	Q2	Q3	P90	P95	Max.
1	350.94	106.4	165.48	187.47	204.73	287.03	347.22	412.72	474.74	503.13	699.77
2	183.15	64.74	82.22	103.16	111.54	135.97	169.11	226.16	273.16	290.75	402.48
3	90.36	39.98	23.68	37.27	44.78	63.89	80.56	110.44	138.4	172.66	215.47
4	185.31	57.91	49.64	103.28	130.3	141.85	182.75	226.25	259.89	288.14	339.03
5	202.41	65.57	70.2	101.44	118.73	146.31	205.89	262.88	283.76	308.44	324.33
6	196.3	86.61	56.86	60.17	82.15	124.12	194.6	254.54	304.68	338.32	418.86
7	331.97	93.72	163.51	189.62	205.01	275.37	320.87	393.45	453.12	498.93	595.27
8	191.71	78.07	66.63	78.84	101.3	140.76	179.19	246.16	288.3	346.65	393.57
9	136.03	46.46	51.5	56.27	74.19	106.91	140.67	159.48	195.44	231.21	235.28
10	112.34	50.83	28.47	39.3	53.05	70.11	107.99	148.77	171.13	202.67	243.3
11	142.39	62.1	49.93	59.92	80.63	99.96	131.32	178.12	218.76	232.3	375.69
12	248.13	92.12	78.75	118.57	149.1	175.04	232.23	306.85	382.61	408.09	483.37
13	240.08	102.67	63.09	104.8	115.3	168.6	223.59	280.56	404.84	440.92	450.68
14	250.82	94.65	112.44	145.73	152.17	164.83	242.99	314.77	414.58	433.72	451.72
15	184.23	88.12	63.37	776.73	98.72	117.53	162.51	236.09	311.58	399.33	408.34
16	160.28	58.98	70.74	87.1	102.71	116.21	148.43	190.56	209.12	311.89	336.28
17	425.87	122.97	234.32	277.31	286.75	324.3	421.82	509.94	605.4	656.89	699.13
18	429.69	141.82	168.16	235.86	271.01	328.38	398.95	529.04	663.58	678.1	685.33
19	396.47	108.61	136.22	246.69	284.49	306.29	400.37	499.25	552.27	559.62	569.92
20	360.78	119.4	165.48	182.12	209.09	274.19	339.62	430.57	516.4	567.54	668.45
21	270.34	97.56	112.01	129.35	146.65	205.15	260.41	320.4	384.52	426.72	602.93
22	310.56	97.63	177.17	182.5	195.95	222.7	293.26	391.19	457.1	461.5	467.1
23	325.24	90.8	193.69	211.39	224.2	252.41	317.99	395.04	425.51	509.82	566.81
24	362.36	125.85	120.02	182.5	217.62	286.3	338.62	437.26	539.2	629.28	681.75
25	357.27	124.13	103.93	144.8	174.86	272.8	367.82	460	494.39	534.46	654.39
26	219.79	54.94	112.44	129.53	142.75	184.07	215.02	260.96	303.66	311.82	323.91
27	275.79	92.17	153.17	165.98	181.51	216.12	254.99	323.44	403.53	471.93	577.04
28	321.91	100.26	147.2	163.69	207.46	245.87	311.03	384.13	457.05	501.43	569.38
29	294.95	103.95	100.03	119.52	154.23	214.97	293.18	382.32	412.4	463.76	490.42
